# Pip5k1γ promotes anabolism of nucleus pulposus cells and intervertebral disc homeostasis by activating CaMKII‐Ampk pathway in aged mice

**DOI:** 10.1111/acel.14237

**Published:** 2024-06-05

**Authors:** Mingjue Chen, Feiyun Li, Minghao Qu, Xiaowan Jin, Tailin He, Shuangshuang He, Sheng Chen, Qing Yao, Lin Wang, Di Chen, Xiaohao Wu, Guozhi Xiao

**Affiliations:** ^1^ Department of Biochemistry, School of Medicine, Shenzhen Key Laboratory of Cell Microenvironment, Guangdong Provincial Key Laboratory of Cell Microenvironment and Disease Research Southern University of Science and Technology Shenzhen China; ^2^ School of Medicine Southern University of Science and Technology Shenzhen China; ^3^ Southern University of Science and Technology Hospital Shenzhen China; ^4^ Department of Orthopaedics, Union Hospital, Tongji Medical College Huazhong University of Science and Technology Wuhan China; ^5^ Research Center for Human Tissues and Organs Degeneration, Shenzhen Institutes of Advanced Technology Chinese Academy of Sciences Shenzhen China; ^6^ Division of Immunology and Rheumatology Stanford University Stanford California USA; ^7^ VA Palo Alto Health Care System Palo Alto California USA

**Keywords:** aging, Ampk, CaMKII, disc degeneration, metformin, nucleus pulposus, Pip5k1γ

## Abstract

Degenerative disc disease (DDD) represents a significant global health challenge, yet its underlying molecular mechanisms remain elusive. This study aimed to investigate the role of type 1 phosphatidylinositol 4‐phosphate 5‐kinase (Pip5k1) in intervertebral disc (IVD) homeostasis and disease. All three Pip5k1 isoforms, namely Pip5k1α, Pip5k1β, and Pip5k1γ, were detectable in mouse and human IVD tissues, with Pip5k1γ displaying a highest expression in nucleus pulposus (NP) cells. The expression of Pip5k1γ was significantly down‐regulated in the NP cells of aged mice and patients with severe DDD. To determine whether Pip5k1γ expression is required for disc homeostasis, we generated a *Pip5k1γ* ^*fl*/*fl*^; *Aggrecan* ^*CreERT2*^ mouse model for the conditional knockout of the *Pip5k1γ* gene in aggrecan‐expressing IVD cells. Our findings revealed that the conditional deletion of Pip5k1γ did not affect the disc structure or cellular composition in 5‐month‐old adult mice. However, in aged (15‐month‐old) mice, this deletion led to several severe degenerative disc defects, including decreased NP cellularity, spontaneous fibrosis and cleft formation, and a loss of the boundary between NP and annulus fibrosus. At the molecular level, the absence of Pip5k1γ reduced the anabolism of NP cells without markedly affecting their catabolic or anti‐catabolic activities. Moreover, the loss of Pip5k1γ significantly dampened the activation of the protective Ampk pathway in NP cells, thereby accelerating NP cell senescence. Notably, Pip5k1γ deficiency blunted the effectiveness of metformin, a potent Ampk activator, in activating the Ampk pathway and mitigating lumbar spine instability (LSI)‐induced disc lesions in mice. Overall, our study unveils a novel role for Pip5k1γ in promoting anabolism and maintaining disc homeostasis, suggesting it as a potential therapeutic target for DDD.

## Graphical abstract

This study identifies the lipid kinase Pip5k1γ as a crucial factor for maintaining intervertebral disc homeostasis, revealing its significant down‐regulation in aged and severely degenerated discs. Conditional deletion of Pip5k1γ in mice leads to advanced disc degeneration, underscoring its importance in extracellular matrix anabolism and the activation of the Ampk pathway. These findings suggest Pip5k1γ as a potential therapeutic target for degenerative disc disease.

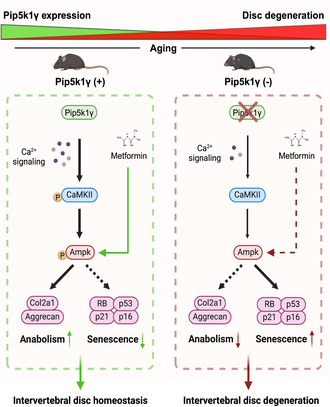

**Abbreviations**
Adamts5: ADAM metallopeptidase with thrombospondin type 1 motif 5
ADP: adenosine diphosphate
AF: annulus fibrosis
AMP: adenosine monophosphate
AMPK: adenosine monophosphate‐activated protein kinase
CA: constitutively active
CaMK: calcium–calmodulin‐dependent protein kinase
CEP: cartilaginous endplates
cKO: conditional knockout
Col2a1: collagen type II alpha 1 chain
CREB: cAMP response element‐binding protein
Dag: diacylglycerol
DDD: degenerative disc disease
ECM: extracellular matrix
EDTA: ethylenediaminetetraacetic acid
ERK: extracellular signal‐regulated kinase
H&E: hematoxylin and eosin
HIF: hypoxia inducible factor
IF: immunofluorescent
Ip3: inositol 1,4,5‐triphosphate
IVD: intervertebral disc
KD: kinase‐dead
LBP: low back pain
LKB1: liver kinase B1
LSI: lumbar spine instability
MAPK: mitogen‐activated protein kinase
Mmp13: matrix metallopeptidase 13
MSCs: mesenchymal stem cells
mTOR: mammalian target of rapamycin
NF‐κB: nuclear factor kappa B
NP: nucleus pulposus
PGC1α: peroxisome proliferator‐activated receptor‐g co‐activator 1 alpha
Pip2: phospholipid phosphatidylinositol 4,5‐bisphosphate
Pip5k1: type 1 phosphatidylinositol 4‐phosphate 5‐kinase
Prx1: paired‐related homeobox protein 1
RT: room temperature
RT‐PCR: real‐time polymerase chain reaction
TAM: tamoxifen
Timp‐1: metalloproteinase‐1
Timp‐3: metalloproteinase‐3
TUNEL: terminal deoxynucleotidyl transferase‐mediated nick‐end labeling
YLDs: years lived with disability

## 1 INTRODUCTION

The intervertebral discs are weight‐bearing structures that connect the vertebral bodies, providing cushioning and flexibility to the spine column. The structure of intervertebral discs comprises three major components, including the nucleus pulposus (NP), annulus fibrosus (AF), and cartilaginous endplates (CEP; Lyu et al., 2019). During aging, intervertebral discs continuously undergo spontaneous degradation. This degradation may cause disc rupture or herniation, leading to a chronic condition known as “degenerative disc disease (DDD)” (Buckwalter, 1995). The pathological features of DDD can be characterized by progressive loss of NP cells and extracellular matrix (ECM), decreased water content, tears in the AF, inflammation, degenerative fibrillation, and osteophyte formation (Maidhof et al., 2014; Wang et al., 2012; Wu et al., 2014). Aging introduces a complex mix of biomolecular damage, abnormal cellular reactions, and structural and functional losses, exemplified by oxidative and inflammatory stresses that impair the ECM and induce cellular senescence (Vo et al., 2013, 2016; Yurube et al., 2023). Alterations in key cellular signaling pathways, including the nuclear factor kappa B (NF‐κB) pathway, the mitogen‐activated protein kinase (MAPK) pathway, the hypoxia inducible factor (HIF) pathway, and the mammalian target of rapamycin (mTOR) pathway, are involved in these pathological processes (Chao‐Yang et al., 2021; Chen, Wu, et al., 2022; Li et al., 2021; Shi et al., 2023; Yurube et al., 2023). Recent findings also highlight the importance of intercellular crosstalk among NP cells, AF progenitors, and immune cells (Wang et al., 2023), adding a layer of complexity to the understanding of DDD pathology.

DDD is a leading cause of low back pain (LBP), which affects more than 600 million people worldwide and causes tremendous economic and healthcare burdens (Chen, Chen, et al., 2022). It has been reported that, from 1990 to 2019, the incidence and prevalence of LBP had substantially increased, and the LBP was the leading cause of years lived with disability (YLDs) in 2019 globally (Chen, Chen, et al., 2022). It is estimated that the annual direct costs per population for LBP range from $2.48 billion to $2.80 billion and the pooled direct costs per patient for LBP are $9231 (95% confidence interval, $7126.71–$25,588.9; Fatoye et al., 2023). However, current therapies for disc degeneration, such as anti‐inflammatory medications, conservative treatments, and surgical interventions, can only provide short‐term pain relief without slowing down the underlying structural deterioration (Peng & Li, 2022). Meanwhile, the development of new disease‐modifying drugs for delaying disc degeneration is still limited in part due to the poorly characterized molecular mechanism of this disease. Thus, a better understanding of the pathological mechanisms underlying the initiation, development, and progression of disc degeneration is urgently needed.

Cellular senescence is a permanent state of growth arrest, where cells are incapable of proliferating, even under conditions that would normally stimulate their proliferation (di Micco et al., 2021). This phenomenon is a critical aging mechanism that manifests across a variety of organs and tissues, notably within the musculoskeletal system (Khosla et al., 2022). Excessive cellular senescence has been documented in several musculoskeletal disorders, including osteoporosis (Farr et al., 2017; Pignolo et al., 2021), osteoarthritis (Chen, Gong, et al., 2022; Liu et al., 2022), and disc degeneration (Novais et al., 2021; Yurube et al., 2023). A common hallmark of cellular senescence is oxidative stress, and several key signaling molecules, such as the adenosine monophosphate (AMP)‐activated protein kinase (AMPK; Han et al., 2016), calcium–calmodulin‐dependent protein kinase (CaMK; Han et al., 2016), extracellular signal‐regulated kinase (ERK; Zou et al., 2019), and cAMP response element‐binding protein (CREB; Hansen 3rd & Zhang, 2013), have been identified as regulators of this process. Emerging research reveals that senolytic drugs, which specifically target senescent cells, could effectively slow the progression of degenerative diseases (Novais et al., 2021), positioning the senescent pathway as a promising therapeutic target for treating these diseases. Nonetheless, the molecular mechanisms by which cellular senescence contributes to disc denegation are still poorly understood.

Type 1 phosphatidylinositol 4‐phosphate 5‐kinases (Pip5k1s), including Pip5k1α, Pip5k1β, and Pip5k1γ, are a class of lipid kinases responsible for the biosynthesis of phospholipid phosphatidylinositol 4,5‐bisphosphate (Pip2; Funakoshi et al., 2011; Heck et al., 2007). The Pip2 can act as a second messenger itself or as a precursor molecule to derive other second messengers, such as inositol 1,4,5‐triphosphate (Ip3) and diacylglycerol (Dag; Nagata & Nozawa, 1992). Pip5k1s play important roles in the regulation of a series of fundamental cellular processes, including the formation of focal adhesion, cell adhesion and migration, cellular trafficking, calcium flux, energy metabolism, and signaling transduction (Di Paolo et al., 2002, 2004; Huang et al., 2022; Ling et al., 2003; Nader et al., 2016; Rodriguez et al., 2012; Schill et al., 2014; Schramp et al., 2011; Xue et al., 2019). Interestingly, recent studies suggest that Pip5k1γ plays an essential role in controlling skeletal development and homeostasis. Zhu and coworkers showed that maintaining a proper level of Pip5k1γ protein is critical for osteoclast formation and functions (Zhu et al., 2013). Results from our group revealed that the Pip5k1γ expression in paired‐related homeobox protein 1 (Prx1)‐expressing cells (primarily the mesenchymal stem cells, MSCs) regulates the bone mass by modulating the bone remodeling in adult mice (Yan et al., 2022). Our more recent study showed that Pip5k1γ exerted important functions in chondrocytes and its loss in these cells induced multiple osteoarthritic lesions in the knee joints of aged mice (Qu et al., 2022). So far, it is not known whether Pip5k1γ is expressed in intervertebral discs and whether it is involved in the regulatory mechanisms of disc homeostasis and disease.

In this study, we find that Pip5k1γ is predominately expressed in the NP cells of intervertebral discs, which is dramatically downregulated in degenerative discs in human DDD patients and aged mice. Conditional deletion of Pip5k1γ expression in aggrecan‐expressing cells reduces anabolism without accelerating catabolism, in NP cells, resulting in DDD‐like phenotypes in aged mice. Pip5k1γ modulates the proliferation, survival and senescence of NP cells through in part regulation of the CaMKII‐Ampk signaling pathway in NP cells.

## 2 METHODS AND MATERIALS

### 2.1 Human NP samples

Human NP samples were collected from 16 DDD patients who underwent unilateral biportal endoscopic discectomy surgery (Table S1). The disease stages of these patients were evaluated by an MRI‐based Pfirrmann grading system. Briefly, patients with grade II‐III defects were classified as mild DDD and those with grade IV‐V defects were classified as severe DDD. Ethics approval was obtained from the Ethics Committee of Tongji Medical College, Huazhong University of Science and Technology (No. [2021] IEC (134), approval date: March 2, 2021). Informed consent was obtained from each participant enrolled in this study. All human sample research was conducted in accordance with the Declaration of Helsinki.

### 2.2 Animals

The generation of *Pip5k1γ* ^*fl*/*fl*^ mice was previously described (Huang et al., 2022; Loo & Zylka, 2017; Qu et al., 2022; Yan et al., 2022). In this study, the *Pip5k1γ* ^*fl*/*fl*^ mice were first crossed with *Aggrecan* ^*CreERT2*^ transgenic mice. This cross produced *Pip5k1γ* ^*fl*/+^; *Aggrecan* ^*CreERT2*^ offsprings. These *Pip5k1γ* ^*fl*/+^; *Aggrecan* ^*CreERT2*^ heterozygous mice were subsequently interbred to generate *Pip5k1γ* ^*fl*/*fl*^; *Aggrecan* ^*CreERT2*^ mice. At 2 months of age, male *Pip5k1γ* ^*fl*/*fl*^; *Aggrecan* ^*CreERT2*^ mice were intraperitoneally injected with TAM (Sigma‐Aldrich, Cat# T5648; 100 mg/kg body weight) for five continuous days following our previously established protocol (Chen, Wu, et al., 2022; Lai et al., 2022; Qu et al., 2022; Wu, Lai, et al., 2022). Age‐ and sex‐matched *Pip5k1γ* ^*fl*/*fl*^; *Aggrecan* ^*CreERT2*^ mice treated with corn oil (Sigma‐Aldrich, Cat# C8267) were used as controls. LSI surgery was performed in L3‐L5 lumbar discs of 2‐month‐old male mice to induce degenerative lesions as previously described (Wu et al., 2023). To test the therapeutic effects of metformin on LSI‐induced disc degeneration in control and cKO mice, 2‐month‐old *Pip5k1γ* ^*fl*/*fl*^; *Aggrecan* ^*CreERT2*^ male mice were treated with TAM/corn oil, and 1 month later, subjected to LSI or sham operations. One week after surgery, metformin was administered (dissolved in drinking water, 205 mg/kg body weight) for another 2 months. The concentration of metformin and the duration of treatment were referred to a similar mouse study conducted by Li et al (2020). All mice in this study were housed at 20–24°C and exposed to half‐day light and half‐day dark cycles. All animal experiments were approved by the Institutional Animal Care and Use Committee of the Southern University of Science and Technology (No. SUSTC‐JY2020119, approval date: June 23, 2020). The *Pip5k1γ* ^*fl*/*fl*^ genotyping primer sequences: Forward 5′‐AGAACCATGAGCCCTAGGCT‐3′, Reverse 5′‐CCTTCCTCATCCTTCTCCAG‐3′. The *Aggrecan* ^*CreERT2*^ genotyping primer sequences: Forward 5′‐GATCTCCGGTATTGAAACTCCAGC‐3′, Reverse 5′‐GCTAAACATGCTTCATCGTCGG‐3′.

### 2.3 LSI‐induced disc degeneration model

The LSI mouse model was initially developed by Fu et al. in 2021, closely replicating the degenerative processes of intervertebral discs in humans, such as reduced disc height, diminished vacuoles in the NP, ectopic bone formation in the CEP, and fissures in the AF tissues (Fu et al., 2021). To establish this model, mice were anesthetized using isoflurane and positioned prone on the surgical table. The surgical procedure involved locating the L5 vertebra, shaving and disinfecting the lower dorsal skin, and making a longitudinal incision to expose the lumbar spine. The posterior paravertebral muscles were detached from vertebrae to expose the L3‐5 spinous processes; the latter were then resected along with the supraspinous and interspinous ligaments. Sham‐operated mice only underwent the detachment of posterior paravertebral muscles without further manipulation. At the end point of experiment, the L4‐5 discs were harvested for further analysis.

### 2.4 Histology

After sacrifice, mouse L4‐5 lumbar discs were fixed in 4% paraformaldehyde for 48 h and then decalcified in 10% ethylenediaminetetraacetic acid (EDTA) for 21 days. Then, the disc samples were dehydrated, paraffin‐embedded, and cut into 5 μm thick sections according to our established protocols (Cao et al., 2020; Fu et al., 2020; Qin et al., 2022; Wang et al., 2019; Yang et al., 2019). The lumbar disc sections were stained with safranin O and fast green using a commercial kit (Solarbio, Cat# G1371). The severity of degenerative disc defects was quantitatively evaluated using a histological scoring system (Melgoza et al., 2021).

### 2.5 Quantitative IF analyses

The IF staining was performed as previously described (Gao et al., 2021; Qin et al., 2021). Briefly, the lumbar disc sections were deparaffinized in xylene and then rehydrated. The rehydrated sections were treated with citrate buffer (0.1 M, pH 6.0) overnight at 65°C for antigen retrieval, and permeabilized with Immunostaining Permeabilization Solution with Saponin (Beyotime, Cat# P0095) for 10 mins at room temperature (RT). After blocking with Immunol Staining Blocking Buffer (Beyotime, Cat# P0102) for 1 h at RT, the lumbar disc sections were incubated with corresponding primary antibodies (Table S2) overnight at 4°C. Then, the disc sections were washed in 1× PBS with 0.1% Tween 20 and incubated with Goat anti‐Rabbit/Mouse IgG (H + L) Cross‐Adsorbed Secondary Antibody, Alexa Fluor 488 (Invitrogen, Cat# A‐11008) for 1 h at RT. After washing in PBS, the sections were mounted with Mounting Medium with DAPI (Abcam, Cat# ab104139). The fluorescent signals were acquired using Zeiss LSM 980 confocal Microscope and analyzed with Image J software. The percentage of signal‐positive cells was calculated by normalizing the count of these cells to the total cell number (identified by DAPI staining).

### 2.6 Cell experiments

The NP cell line used in this study was generated and gifted by Dr. Di Chen (Shenzhen Institutes of Advanced Technology; Chen et al., 2020; Chen, Wu, et al., 2022; Oh et al., 2016). The maintenance of NP phenotypes was validated by real‐time PCR assays (Figure S3). The NP cells were cultured in DMEM (Corning, Cat# 10‐013‐CVR) containing 10% FBS (Gibco, Cat# 10099–141) and 1% penicillin–streptomycin (Hyclone, Cat#SV30010) at 37°C under normal oxygen conditions supplied with 5% CO_2_. To investigate the effects of Pip5k1γ knockdown in NP cells in vitro, the cells were transfected with negative control siRNA or Pip5k1γ ‐targeting siRNA using Lipofectamine™ RNAiMAX transfection Reagent (Invitrogen, Cat# 13778150). The siRNA/reagent complex was applied to the NP cells 24 h post‐seeding in the culture dish. To test the effects of CaMKII activation on Pip5k1γ loss‐induced cellular phenotypes, the NP cells were transfected Pip5k1γ siRNA in combination with plasmids expressing either a kinase‐dead *CaMKII* (IGE Biotechnology, K42R) gene or constitutively activated *CaMKII* (IGE Biotechnology, T286D) gene using Lipofectamine™ 3000 Transfection Reagent (Invitrogen, L3000015). The transfection procedures were conducted following manufactural instructions. The Pip5k1γ siRNA sequences: (#1) 5′‐ GCGUGCAGUCUGGUGGCAATT‐3′, 3′‐ UUGCCACCAGACUGCACGCTT‐5′; (#2) 5′‐ GCUUCUAUGCAGAGCGCUUTT‐3′, 3′‐ AAGCGCUCUGCAUAGAAGCTT‐5′; (#3) 5′‐ GCUGGACUCCGACACCUUUTT‐3′, 3′‐ AAAGGUGUCGGAGUCCAGCTT‐5′. All in vitro experiments were conducted with at least three biological replications.

### 2.7 Real‐time PCR

The real‐time PCR analysis was carried out in accordance with our established protocol (Yan et al., 2022). The sequences of the primers used are as follows: For *Pip5k1γ*, the forward primer is GGATGCGTCGGGAGAGACTA, and the reverse primer is AGGTCTTGAAGCGGAAATCCT; for *Acan*, the forward primer is AGGATGGCTTCCACCAGTGC, and the reverse primer is TGCGTAAAAGACCTCACCCTCC; for *Col2a1*, the forward primer is CCTGGACCCCGTGGCAGAGA, and the reverse primer is CAGCCATCTGGGCTGCAAAG; for *Mmp13*, the forward primer is GCAGCTCCAAAGGCTACAA, and the reverse primer is CATCATCTGGGAGCATGAAA; for *Adamts5*, the forward primer is TGGAGTGTGTGGAGGGGATA, and the reverse primer is CGGACTTTTATGTGGGTTGC; for *Timp‐1*, the forward primer is GCAACTCGGACCTGGTCATAA, and the reverse primer is CGGCCCGTGATGAGAAACT; for *RB*, the forward primer is TGCATCTTTATCGCAGCAGTT, and the reverse primer is GTTCACACGTCCGTTCTAATTTG; for *p53*, the forward primer is GCGTAAACGCTTCGAGATGTT, and the reverse primer is TTTTTATGGCGGGAAGTAGACTG; for *p21*, the forward primer is CCTGGTGATGTCCGACCTG, and the reverse primer is CCATGAGCGCATCGCAATC; for *p16*, the forward primer is CTTCACCAACGCCCCGAACAC, and the reverse primer is CGGGAGAGGGTGGTGGGGTC; for *Ampk*, the forward primer is GTCAAAGCCGACCCAATGATA, and the reverse primer is CGTACACGCAAATAATAGGGGTT; for *CaMKII*, the forward primer is GCCTACATCCGCATCACTCA, and the reverse primer is GGCCTGGTCCTTCAATGGG; for *Vimentin*, the forward primer is CAGACAGGATGTTGACAATGC, and the reverse primer is GCTCCTGGATCTCTTCATCG; for *Laminin*, the forward primer is AGCTTTGTGATGGACAGTGG, and the reverse primer is CTTCTAGCCGCAGTGTGTTC; for *Basp1*, the forward primer is CGTCCAAGGAGACGCCCG, and the reverse primer is TGCTCGGAGCTGGCGACG; for *Tbxt*, the forward primer is AGAATGAGGAGATTACGGCCC, and the reverse primer is ATTGGGAATATCCCGGCTGC; for *CD24*, the forward primer is TGCTTCTGGCACTGCTCCTAC, and the reverse primer is GGTGGTAGCATTAGTTGGATTTGG; for Gapdh, the forward primer is AGGTCGGTGTGAACGGATTTG, and the reverse primer is TGTAGACCATGTAGTTGAGGTCA.

### 2.8 Western blotting analyses

The western blot analyses were performed as previously described (Gao et al., 2022; Lei et al., 2020; Wu et al., 2015; Zhu et al., 2020). The primary antibodies used in this study are shown in Table S2. Gapdh is utilized as a loading control (Yan et al., 2022; Yurube et al., 2011). In Pip5k1γ siRNA knockdown experiments, it was validated that the mRNA and protein levels of Gapdh remained unchanged at 24 h after Pip5k1γ siRNA treatment (Table S3).

### 2.9 TUNEL staining

The TUNEL staining was performed using the One Step TUNEL Apoptosis Assay Kit (Red Fluorescence; Beyotime, Cat# C1090) as previously described (Lei et al., 2020; Qu et al., 2022).

### 2.10 Cytosol Ca^2+^ assay

The cytoplasmic Ca^2+^ levels were detected using the Ca^2+^‐sensitive Fluorescent Dye Fluo‐4/AM (Yeasen, Cat#40704ES50) as previously described (Yan et al., 2022).

### 2.11 Statistical analysis

Statistical analyses were conducted using the Prism GraphPad software. Results are presented as mean ± standard deviation (SD), with the sample size of each experiment indicated in the corresponding figure legends. The normality of data distribution was assessed using the Kolmogorov–Smirnov (K‐S) test. A two‐tailed unpaired Student's *t* test was employed to compare the statistical difference between two groups. In comparisons between multiple groups, a one‐way ANOVA followed by Tukey's post hoc test was used. A two‐way ANOVA test was applied to analyze differences between groups with two independent variables. Statistical significance was set at *p* < 0.05.

## 3 RESULTS

### 3.1 Reduced Pip5k1γ expression in NP cells in discs from aged mice and human DDD patients

**FIGURE 1:**
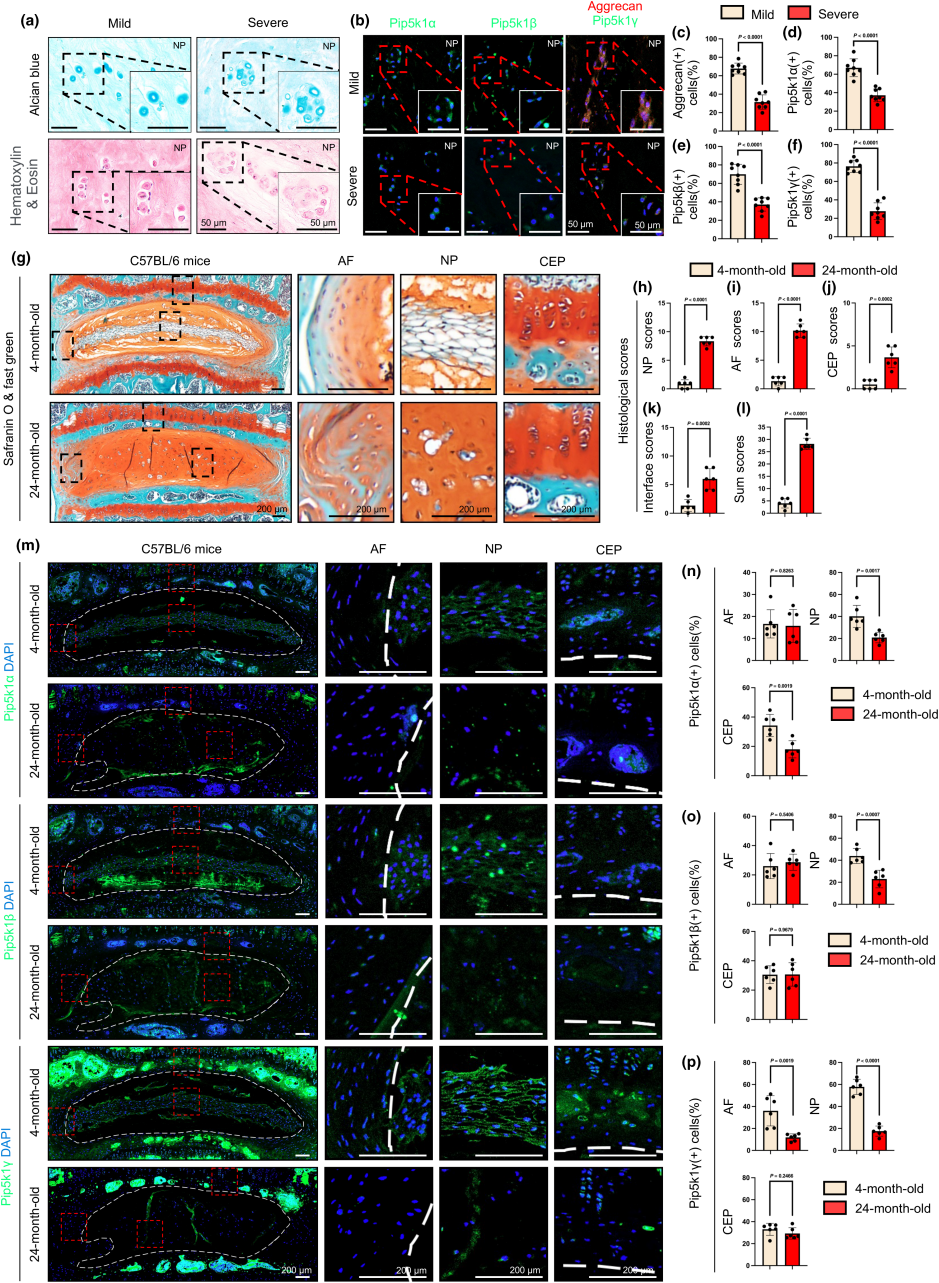
Decreased expression of Pip5k1γ protein in lumbar discs of DDD patients and aged mice. (a) Alcian blue (upper panels) and H&E (lower panels) of NP specimens from patients with mild or severe DDD. Scale bar, 50 μm. (b–f) Quantitative IF analyses of aggrecan, Pip5k1α, Pip5k1β, and Pip5k1γ in NP sections. Scale bar: 50 μm. *N* = 8 per group. (g) Safranin O and fast green staining of L4‐5 lumbar disc sections from 4‐ and 24‐month‐old male C57BL/6 mice. Scale bar, 200 μm. Higher magnification images of the AF, NP, and CEP areas (black dashed boxes) were shown in the right panels. (h–l) Histological scores quantifying the severity of disc degeneration, including NP scores, AF scores, CEP scores, Interface scores, and Sum scores. *N* = 6 for each group. (m) IF staining showing the expression of Pip5k1α, Pip5k1β, and Pip5k1γ proteins (green signals) in L4‐5 lumber discs from 4‐ and 24‐month‐old male C57BL/6 mice. Higher magnification images of the AF, NP, and CEP areas (red dashed boxes) are shown in the right panels. White dashed lines indicate the boundary between AF and NP. Scale bar, 200 μm. (n–p) Percentages of Pip5k1α‐, Pip5k1β‐, and Pip5k1γ‐positive cells in AF, NP, and CEP tissues, respectively. *N* = 6 for each group. Results are expressed as mean ± standard deviation (SD). *p*‐values for individual analyses are provided. AF, annulus fibrosus; CEP, cartilaginous endplates; NP, nucleus pulposus.

We initially investigated the expression levels of three Pip5k1 isoforms, namely Pip5k1α, Pip5k1β, and Pip5k1γ, at different disease stages in DDD patients. NP tissues were collected though unilateral biportal endoscopic discectomy from patients diagnosed with mild or severe DDD, as determined by an MRI‐based Pfirrmann grading system (Pfirrmann et al., 2001). Their MRI images were independently analyzed multiple times by two researchers in a blinded manner. The age, sex, Pfirrmann grades, and segment levels of included patients were described in Table S1. The collected NP tissues were subjected to alcian blue and hematoxylin and eosin (H&E) staining to evaluate the degree of degeneration (Figure 1a). Compared with the mild group, NP tissues from the severe DDD group exerted a lower cellularity, loss of alcian blue stain, and the appearance of abnormal cell clustering (Figure 1a). The protein expressions of Pip5k1α, Pip5k1β, Pip5k1γ, and anabolic ECM protein aggrecan were determined by immunofluorescent (IF) analysis. The numbers of signal‐positive cells and total cells were quantified using an Image J software. Then, the percentage of signal‐positive cells was calculated by normalizing the count of signal‐positive cells to the total number of cells. The results revealed that Pip5k1α, Pip5k1β, and Pip5k1γ were all detectable in human NP tissues (Figure 1b). The percentages of these Pip5k1 proteins and aggrecan were significantly decreased in NP tissues from the severe DDD group compared to those from the mild group (Figure 1b–f). Next, we collected the L4‐5 lumbar discs from male C57BL/6 mice at 4 and 24 months of age and performed histology and IF analysis. Results from safranin O and fast green staining showed that the NP compartments were enriched with vacuolated cells and surrounded by highly organized AF layers in 4‐month‐old mice (Figure 1g). In contrast, the lumbar discs from 24‐month‐old mice displayed severe degenerative defects. These included the complete loss of vacuolated NP cells, the loss of boundary between AF and NP, the appearance of hypertrophic cells, ECM fibrosis and fissures, and disorganized AF layers (Figure 1g). The severity of these degenerative defects was quantified using a histological scoring system (Melgoza et al., 2021). Briefly, this scoring system involves evaluating a series of key histopathological features from NP, AF, CEP, and their interfaces, with each feature being categorized and scored to accurately quantify mouse intervertebral disc pathologies. The results revealed that the histopathological scores of aged discs were all drastically higher than those of discs from healthy adult mice (Figure 1h–l). Consistent with results from humans, IF staining showed that all three Pip5k1 isoforms were detectable in mouse disc tissues, including the NP, AF, and CEP. Importantly, when compared with adult discs, the expression levels of Pip5k1γ were dramatically down‐regulated in NP and AF tissues of aged mice (Figure 1m–p).

### 3.2 Deleting Pip5k1γ expression in aggrecan‐expressing cells accelerates disc degeneration in aged mice

**FIGURE 2:**
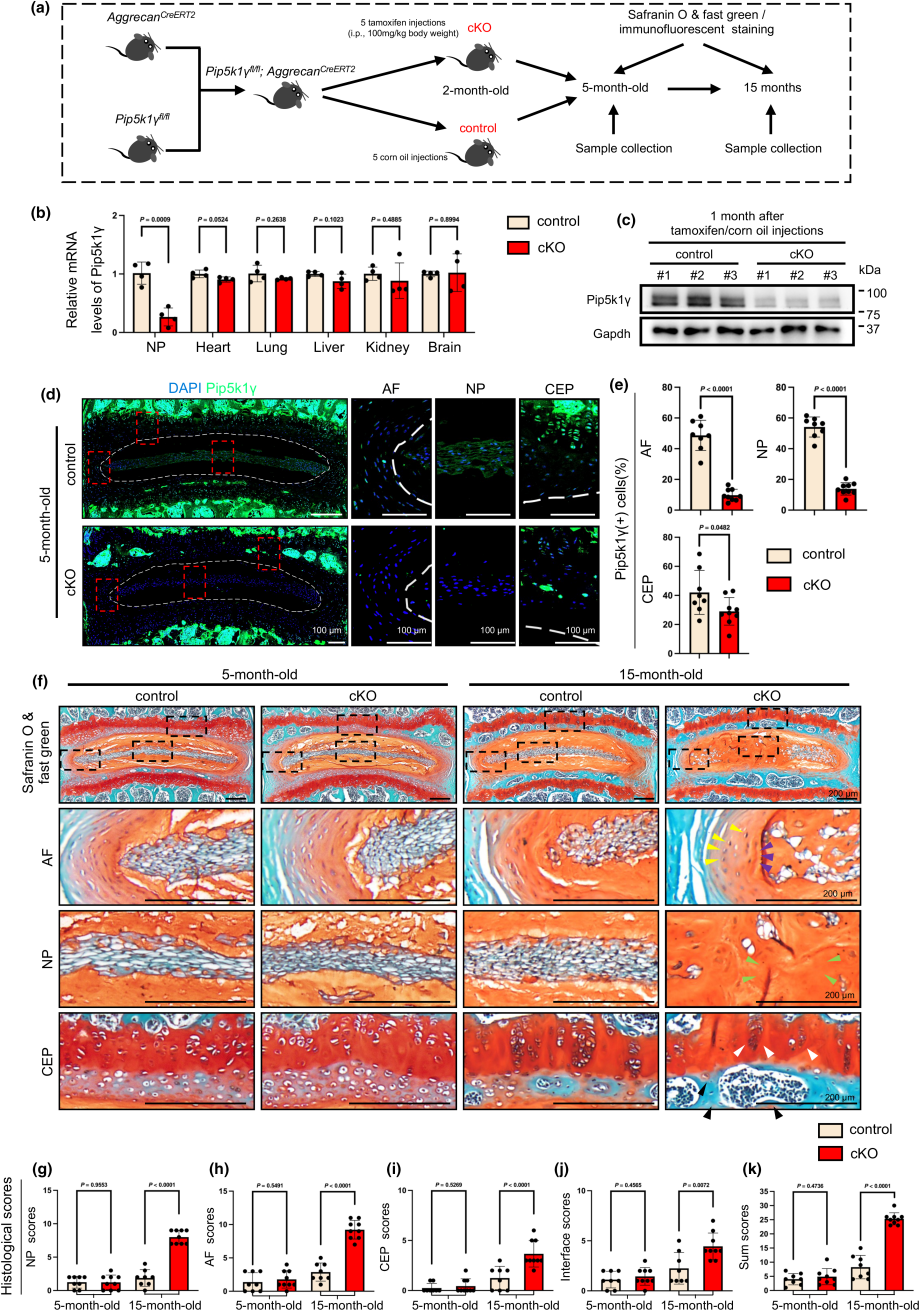
Effects of Pip5k1γ deletion on lumbar disc structure in adult and aged mice. (a) A schematic diagram illustrating the experimental design. Two‐month‐old male *Pip5k1γ* ^*fl*/*fl*^; *Aggrecan* ^*CreERT2*^ mice were treated with TAM for inducible deletion of the *Pip5k1γ* gene in aggrecan‐expressing disc cells (cKO). Age‐ and sex‐matched *Pip5k1γ* ^*fl*/*fl*^; *Aggrecan* ^*CreERT2*^ mice were treated with corn oil as the control group. (b) Real‐time polymerase chain reaction (RT‐PCR) analysis of *Pip5k1γ* mRNA levels in NP, heart, lung, liver, kidney, and brain tissues from control or cKO mice at 1 month after TAM or corn oil injections. *N* = 4 for each group. (c) Protein levels of Pip5k1γ in isolated NP tissues from control or cKO mice at 1 month after TAM or corn oil injections. *N* = 3 for each group. (d) Representative IF staining showing the Pip5k1γ expression in lumbar disc sections from control and cKO mice at 5 months of age. Higher magnification images of the AF, NP, and CEP areas (red dashed boxes) are shown in the right panels. Scale bar: 100 μm. (e) Percentages of Pip5k1γ‐positive cells in the AF, NP, and CEP tissues. *N* = 8 for control and *N* = 9 for cKO. (f) Safranin O and fast green staining showing the structure of lumber discs of control and cKO mice at 5 or 15 months of age. Higher magnification images of AF, NP, and CEP areas (black dashed boxes) are shown in lower panels. Scale bar: 200 μm. The green arrowheads indicate the loss of vacuolar NP cells and spontaneous fissures. The yellow arrowheads indicate the appearance of hypertrophic cells. The purple arrowheads indicate the buckling of AF lamellae into NP compartment. The black arrowheads indicate the loss of safranin O staining in CEP. The white arrowheads indicate the decreased cellularity in CEP. (g–k) Quantitative histological scorings evaluating the degenerative disc defects in control and cKO mice. *N* = 8 for control and *N* = 9 for cKO. Results are expressed as mean ± standard deviation (SD). *p*‐values for individual analyses are provided. AF, annulus fibrosus; CEP, cartilaginous endplates; NP, nucleus pulposus.

The above results lead us to hypothesize that Pip5k1γ may play a pivotal role in the pathogenesis of disc degeneration. To explore this possibility, we generated *Pip5k1γ* ^*fl*/*fl*^; *Aggrecan* ^*CreERT2*^ mice by crossing *Pip5k1γ* ^*fl*/*fl*^ mice with *Aggrecan* ^*CreERT2*^ transgenic mice. To induce deletion of *Pip5k1γ* gene in aggrecan‐expressing disc cells (primarily NP, AF, and CEP cells), 2‐month‐old male *Pip5k1γ* ^*fl*/*fl*^; *Aggrecan* ^*CreERT2*^ mice received five daily intraperitoneal injections of tamoxifen (TAM), as illustrated in Figure 2a. These mice are hereafter referred to as cKO (conditional knockout). The dose of TAM (100 mg per kg body weight) and duration (5 days) are sufficient to induce conditional gene knockout in aggrecan‐expressing cells based on our previous experiences (Chen, Wu, et al., 2022; Lai et al., 2022; Qu et al., 2022; Wu, Lai, et al., 2022). Corn oil was utilized as a solvent to dissolve TAM, and an equal volume of corn oil was injected into another batch of age‐ and sex‐matched *Pip5k1γ* ^*fl*/*fl*^; *Aggrecan* ^*CreERT2*^ mice, serving as a control group for the cKO mice. Real‐time polymerase chain reaction (RT‐PCR) and western blotting analyses confirmed a specific deletion of Pip5k1γ in NP tissues of cKO mice at 1 month after TAM injections (Figure 2b,c). To test the effects of Pip5k1γ deletion on disc structural integrity, the L4‐5 lumbar disc samples were harvested at 3 and 13 months after TAM injections and subjected to histological and IF analyses. IF staining results confirmed that the expression of Pip5k1γ protein was essentially abolished, while that of Pip5k1α and Pip5k1β proteins was not markedly affected, in the AF and NP cells of cKO mice at 3 months after TAM injections (Figure 2d,e and Figure S1). Histological analyses revealed that the disc structure and all histological scores were comparable between the control and cKO groups at 3 months after TAM injections (Figure 2f–k). Remarkably, at 13 months after TAM injections, cKO mice exhibited multiple severe degenerative defects in their discs, including the loss of vacuolar NP cells and spontaneous fissures (Figure 2f, green arrowheads), the appearance of hypertrophic cells (Figure 2f, yellow arrowheads), the buckling of AF lamellae into NP compartment (Figure 2f, purple arrowheads), and loss of safranin O staining in CEP (Figure 2f, black arrowheads). Decreased cellularity was also observed across the whole disc compartments, while the CEP thickness was comparable between the cKO and control discs (Figure 2f, white arrowheads). Moreover, quantitative data showed significant increases in the histopathological scores, including NP scores (Figure 2g), AF scores (Figure 2h), CEP scores (Figure 2i), Interface scores (Figure 2j) in cKO mice at 13 months after TAM injections compared to age‐matched control mice. The Sum scores, which combine all the above scores, were markedly higher in the cKO than in control discs at 13 months after TAM injections (Figure 2k). It should be noted that, at this time point, the expression level of Pip5k1α slightly increased, while that of Pip5k1β remained unchanged, in the NP tissues of cKO mice relative to control mice (Figure S1). Collectively, these findings demonstrate that genetic deletion of Pip5k1γ in aggrecan‐expressing cells significantly accelerates disc degeneration in aged mice.

### 3.3 Pip5k1γ deletion reduces ECM anabolism without affecting ECM catabolism in NP cells

**FIGURE 3:**
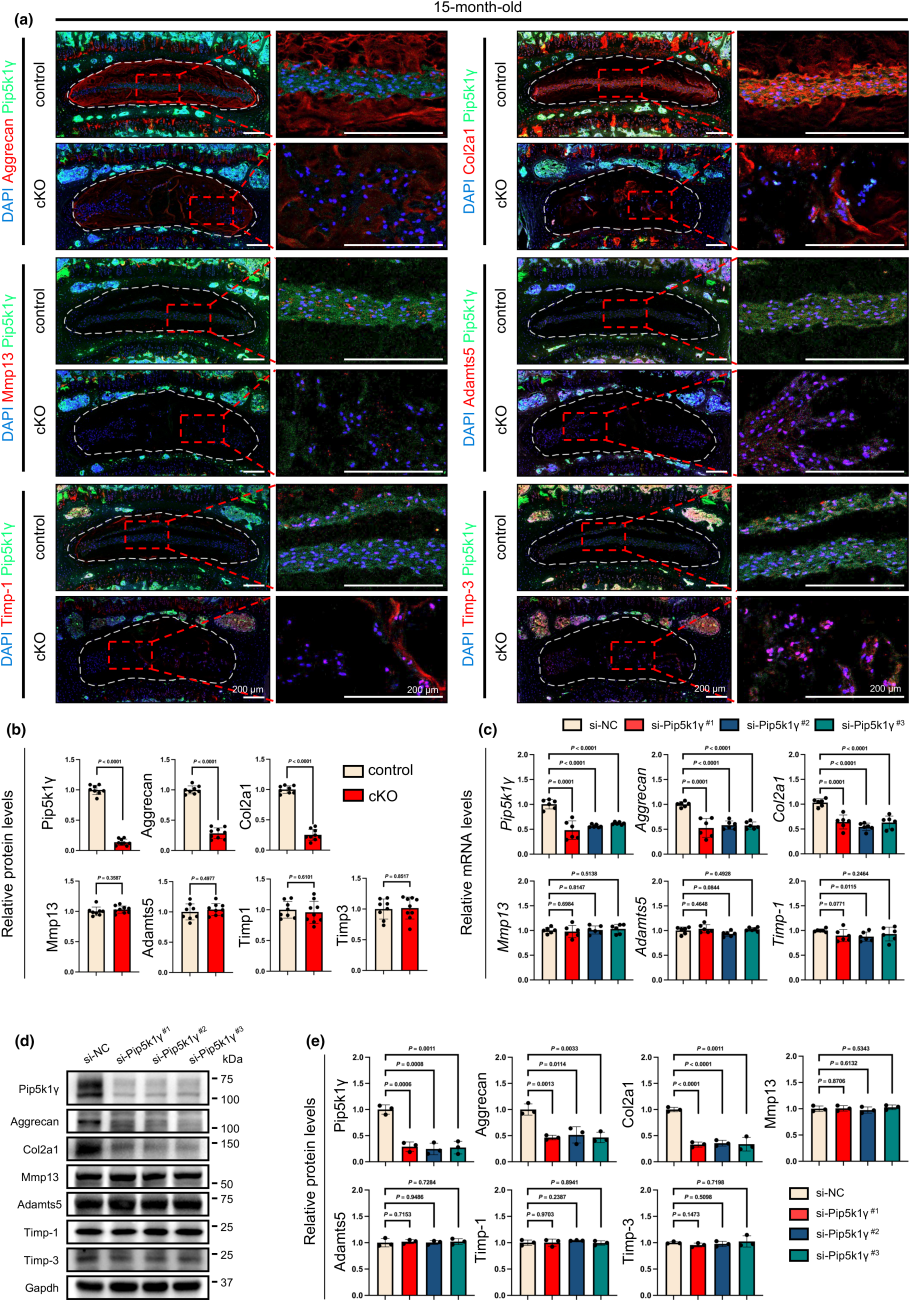
Impact of Pip5k1γ deletion on ECM homeostasis in aged mice. (a) Representative double IF staining images depicting the co‐expression of Pip5k1γ and other key molecules, including aggrecan, Col2a1, Mmp13, Adamts5, Timp1, and Timp3 in lumbar discs of control and cKO mice at 15 months of age. Higher magnification images of the NP areas (red dashed boxes) are presented in the right panels. Scale bar, 200 μm. (b) Quantification of relative protein expression levels for Pip5k1γ, aggrecan, Col2a1, Mmp13, Adamts5, Timp1, and Timp3 in NP tissues of control and cKO discs. *N* = 8 for control and *N* = 9 for cKO. (c) Relative mRNA levels of *Pip5k1γ*, *aggrecan*, *Col2a1*, *Mmp13*, *Adamts5*, and *Timp1* in NP cells were assessed following transfection with negative control siRNA (si‐NC) or Pip5k1γ‐targeting siRNA (si‐Pip5k1γ) for 48 h. Three different siRNA sequences, namely si‐Pip5k1γ^#1^, si‐Pip5k1γ^#2^, si‐Pip5k1γ^#3^, were used to confirm the specificity of Pip5k1γ knockdown in this experiment. *Gapdh* served as a housekeeping gene. *N* = 6 for each group. (d, e) Western blot analysis of NP cells transfected with negative control si‐NC or si‐Pip5k1γ sequences for 48 h. Gapdh was used as a loading control. Quantitative data are displayed in (e). *N* = 3 for each group. Results are presented as mean ± standard deviation (SD). *p*‐values for individual analyses are provided.

The results from IF staining of the lumbar L4‐5 disc sections obtained from 15‐month‐old control and cKO mice revealed that Pip5k1γ loss significantly reduced the expression levels of anabolic ECM proteins, including aggrecan and collagen type II alpha 1 chain (Col2a1), in the NP cells of cKO mice in comparison to control discs (Figure 3a,b). Furthermore, matrix metallopeptidase 13 (Mmp13) and ADAM metallopeptidase with thrombospondin type 1 motif 5 (Adamts5), which are two crucial catabolic ECM enzymes, were expressed at relatively low levels in NP tissues and showed no significant differences between the control and cKO groups (Figure 3a,b). The expression levels of anti‐catabolic factors, including the tissue inhibitor of metalloproteinase‐1 (Timp‐1) and Timp‐3, were also comparable between the control and cKO groups (Figure 3a,b). To validate these in vivo findings, we performed a knockdown of Pip5k1γ expression using three different siRNA sequences in an immortalized NP cell line (Chen et al., 2020; Chen, Wu, et al., 2022; Oh et al., 2016) and found that the Pip5k1γ knockdown substantially reduced the mRNA and protein levels of aggrecan and Col2a1, while those of catabolic and anti‐catabolic factors (Mmp13, Adamts5, Timp1, and Timp3) were not markedly affected by Pip5k1γ siRNAs in NP cells (Figure 3c–e).

### 3.4 Pip5k1γ deletion reduces cellularity in NP tissue by suppressing cell proliferation and promoting cell apoptosis

**FIGURE 4:**
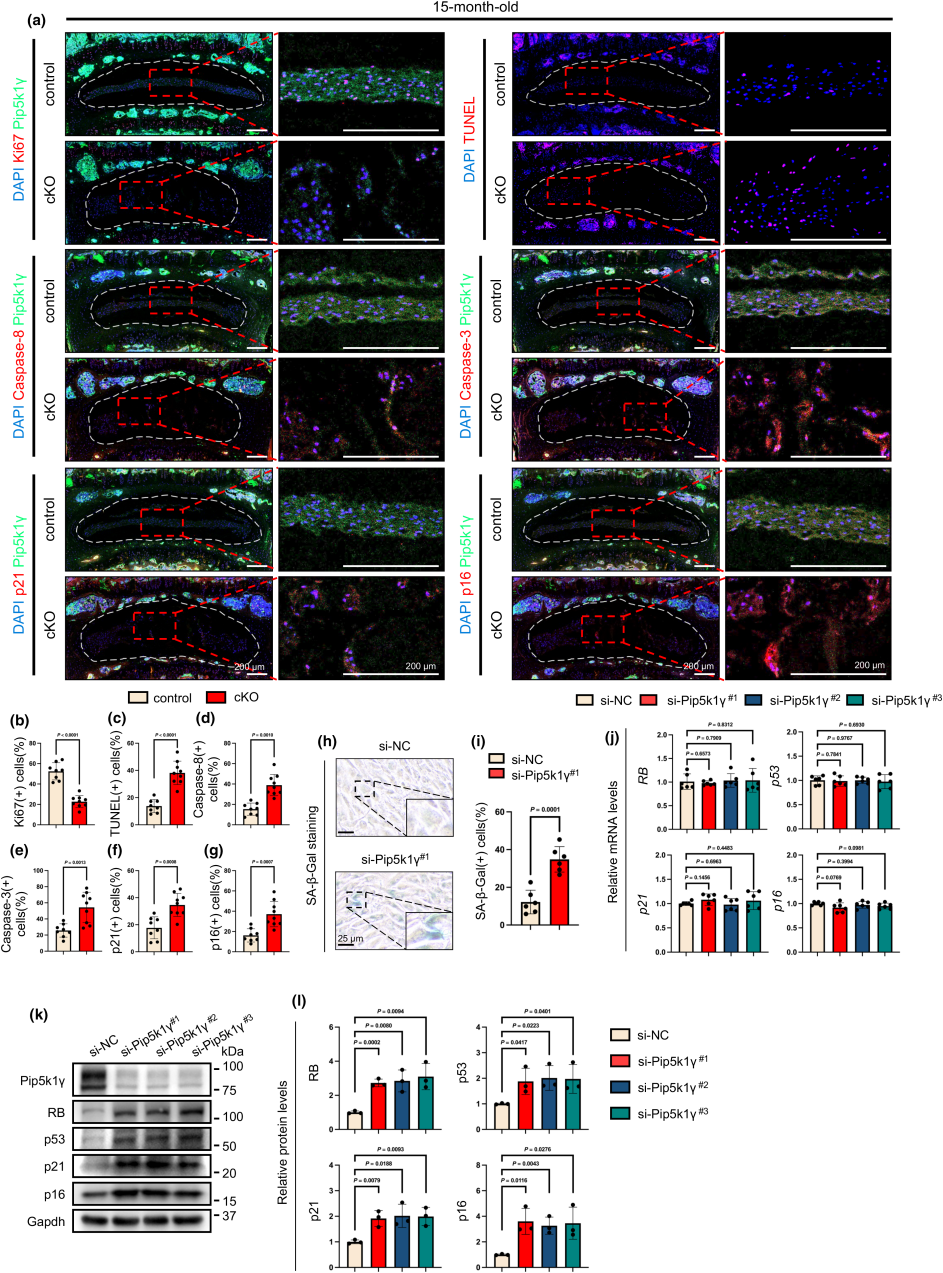
Effects of Pip5k1γ deletion on cell proliferation, apoptosis, and cellular senescence in NP cells. (a) Representative staining showing the co‐expression of Pip5k1γ and cellular proliferation marker Ki67, cellular apoptosis markers caspase 3, and caspase 8, and cellular senescent markers p21 and p16 in lumbar discs from both control and cKO mice at 15 months of age. Terminal‐deoxynucleotidyl transferase‐mediated nick end labeling (TUNEL) staining was performed to determine the apoptotic activity of NP cells. Higher magnification images of the NP areas (red dashed boxes) are presented in the right panels. Scale bar, 200 μm. (b–g) Percentages of Ki67‐, TUNEL‐, caspase‐8‐, caspase‐3‐, p21‐, and p16‐positive cells in NP tissues, respectively. *N* = 8 for control and *N* = 9 for cKO. (h) SA‐β‐Gal staining revealing senescent NP cells (blue signals) after transfection with si‐Pip5k1γ^#1^ for 48 h. si‐NC‐treated NP cells were used as controls. Scale bar, 25 μm. (i) Calculation of the percentages of SA‐β‐Gal‐positive cells in six different areas under the microscope. (j) Relative mRNA levels of *RB*, *p53*, *p21*, and *p16* in NP cells transfected with si‐NC or si‐Pip5k1γ for 48 h. *Gapdh* served as a housekeeping gene. *N* = 6 for each group. (k) Western blotting analysis illustrating the protein levels of Pip5k1γ and senescence markers, including RB, p53, p21, and p16 in NP cells transfected with si‐NC or si‐Pip5k1γ for 48 h. Gapdh was used as an endogenous loading control. (l) Quantitative data of (k). *N* = 3 for each group. Results are expressed as mean ± standard deviation (SD). *p*‐values for individual analyses are provided.

The results from IF staining of disc sections of the two genotypes using an anti‐Ki67 antibody showed that Ki67 was highly expressed in NP tissues of control discs (Figure 4a). However, the percentage of Ki67‐positive cells was significantly decreased in NP areas of control discs when compared with those of cKO discs (Figure 4b). We further performed the terminal deoxynucleotidyl transferase‐mediated nick‐end labeling (TUNEL) staining of disc sections to measure apoptotic cells and found that Pip5k1γ deletion significantly increased the percentage of apoptotic cells (Figure 4a,c). Furthermore, we observed that Pip5k1γ loss increased the percentages of caspase‐3‐ and caspase‐8‐expressing NP cells in cKO versus control discs (Figure 4a,d,e).

### 3.5 Pip5k1γ deletion induces cellular senescence in NP cells

We observed that the absence of Pip5k1γ led to an upregulation in the expression of cellular senescence markers, namely p21 and p16, in NP tissues of cKO mice compared to the control group (Figure 4a,f,g). In addition, the loss of Pip5k1γ resulted in a significant increase in the percentages of SA‐β‐Gal‐positive NP cells in vitro (Figure 4h,i). We further examined the mRNA and protein expressions of cellular senescence markers, namely RB, p53, p21, and p16, in NP cells following transfection with Pip5k1γ siRNAs. Interestingly, although the mRNA levels of these markers were not elevated (Figure 4j), western blotting analyses demonstrated that Pip5k1γ knockdown significantly up‐regulated the protein levels of all senescence markers in NP cells in vitro (Figure 4k,l), suggesting that mechanism(s) of post‐transcription regulation are involved in this regulation. Collectively, these findings demonstrate that the absence of Pip5k1γ induces a cellular senescent phenotype in NP cells.

### 3.6 Pip5k1γ deletion inhibits activation of the CaMKII‐Ampk pathway in NP cells

**FIGURE 5:**
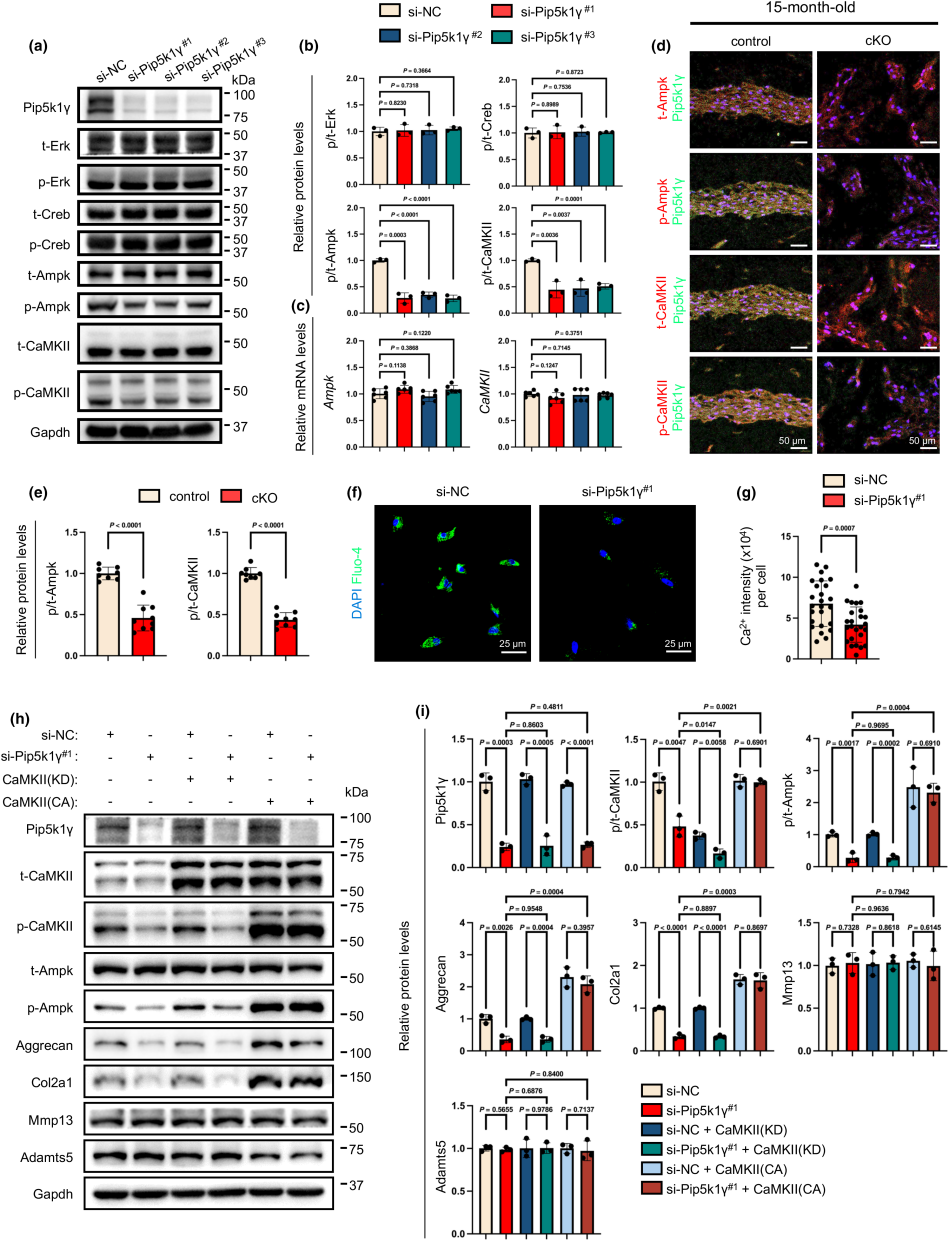
Pip5k1γ deletion inhibits Ampk activation through CaMKII signaling pathway. (a, b) Western blot showing the effects of Pip5k1γ knockdown on expression and activation of key cellular signaling factors, including Erk, Creb, Ampk, CaMK, in NP cells in vitro. Gapdh was used as a loading control. Quantitative data are shown in (b). *N* = 3 for each group. “t” represents total protein, and “p” represents phosphorylated protein. (c) Relative mRNA levels of *Ampk* and *CaMKII* in NP cells following transfection of si‐NC or si‐Pip5k1γ for 48 h. *N* = 6 for each group. (d, e) Representative IF staining of co‐expression of Pip5k1γ with total or phosphorylated Ampk and CaMKII in the NP tissues of control and cKO mice at 15 months of age. Scale bar, 50 μm. Quantitative data are shown in (e). (f, g) Cytosol Ca^2+^ assay. NP cells were treated with si‐NC or si‐Pip5k1γ^#1^ for 48 h and then subjected to Fluo‐4/AM staining. Green signals indicate the cytoplasmic Ca^2+^. Scale bar: 25 μm. The Ca^2+^ fluorescent intensity per cell was analyzed and shown in (g). *N* = 25 for each group. (h, i) NP cells were transfected with si‐NC or si‐Pip5k1γ^#1^ in combination with plasmids expressing a kinase‐dead (KD) or constitutively active (CA) CaMKII for 48 h. Total protein extracts were analyzed by western blotting using antibodies against Pip5k1γ, t‐CaMKII, p‐CaMKII, t‐Ampk, p‐Ampk, aggrecan, Col2a1, Mmp13, and Adamts5. Gapdh served as a loading control. Quantitative data were shown in (i). *N* = 3 for each group. Results are expressed as mean ± standard deviation (SD). *p*‐values for individual analyses are provided.

We further examined the effects of Pip5k1γ knockdown on key cellular signaling pathways, including Erk, Creb, Ampk, and CaMKII, in NP cells in vitro. Pip5k1γ siRNA did not markedly alter the levels of total and phosphorylated protein levels of the Erk and Creb proteins in NP cells (Figure 5a,b). In contrast, Pip5k1γ siRNA treatment drastically reduced the phosphorylation of Ampk and its upstream molecule, CaMKII, in NP cells, without affecting their mRNA levels (Figure 5a–c). In vivo, the deletion of Pip5k1γ caused dramatic reductions in protein expression of phosphorylated Ampk (p‐Ampk) and phosphorylated CaMKII (p‐CaMKII) in NP cells, as revealed by IF staining of disc sections of control and cKO mice (Figure 5d,e). CaMKII is necessary for Ca^2+^ homeostasis and has been reported to induce Ampk activation (Wright et al., 2004; Xie et al., 2015; Zhu et al., 2018). Thus, we assessed whether the deletion of Pip5k1γ could impact cellular Ca^2+^ influx in NP cells. The NP cells were seeded at a density of 2 × 10^5^ cells per well in a 6‐well plate, and 12 h later, transfected with either negative control siRNA (si‐NC) or Pip5k1γ‐targeting siRNA (si‐Pip5k1γ). After 48 h of transfection, cells were harvested and seeded at a density of 2 × 10^5^ cells per dish in confocal dishes and incubate for another 12 h. Then, the intracellular Ca^2+^ intensity was analyzed using a Fluo‐4 kit. Results from Fluo‐4 staining demonstrated that loss of Pip5k1γ reduced the intracellular Ca^2+^ in NP cells in vitro (Figure 5f,g). To investigate whether enforced activation of the CaMKII could reverse the decreases in p‐Ampk and ECM anabolism induced by Pip5k1γ deletion, we transfected NP cells with si‐Pip5k1γ in combination with plasmids expressing either a constitutively active form of CaMKII (CA‐CaMKII) or a kinase‐dead form of CaMKII (KD‐CaMKII). The results indicated that KD‐CaMKII had no effects on the phosphorylated/total protein ratios of Ampk and CaMKII, as well as the anabolic and catabolic ECM proteins in NP cells (Figure 5h,i). In contrast, CA‐CaMKII treatment significantly up‐regulated the phosphorylated/total protein ratios of Ampk and CaMKII and anabolic ECM proteins in Pip5k1γ siRNA‐treated NP cells (Figure 5h,i). Collectively, these results suggest that Pip5k1γ promotes ECM anabolism, at least in part, by activating the CaMKII‐Ampk pathway in NP cells.

### 3.7 Pip5k1γ deletion blunts the therapeutic effects of metformin on instability‐induced disc lesions in mice

**FIGURE 6:**
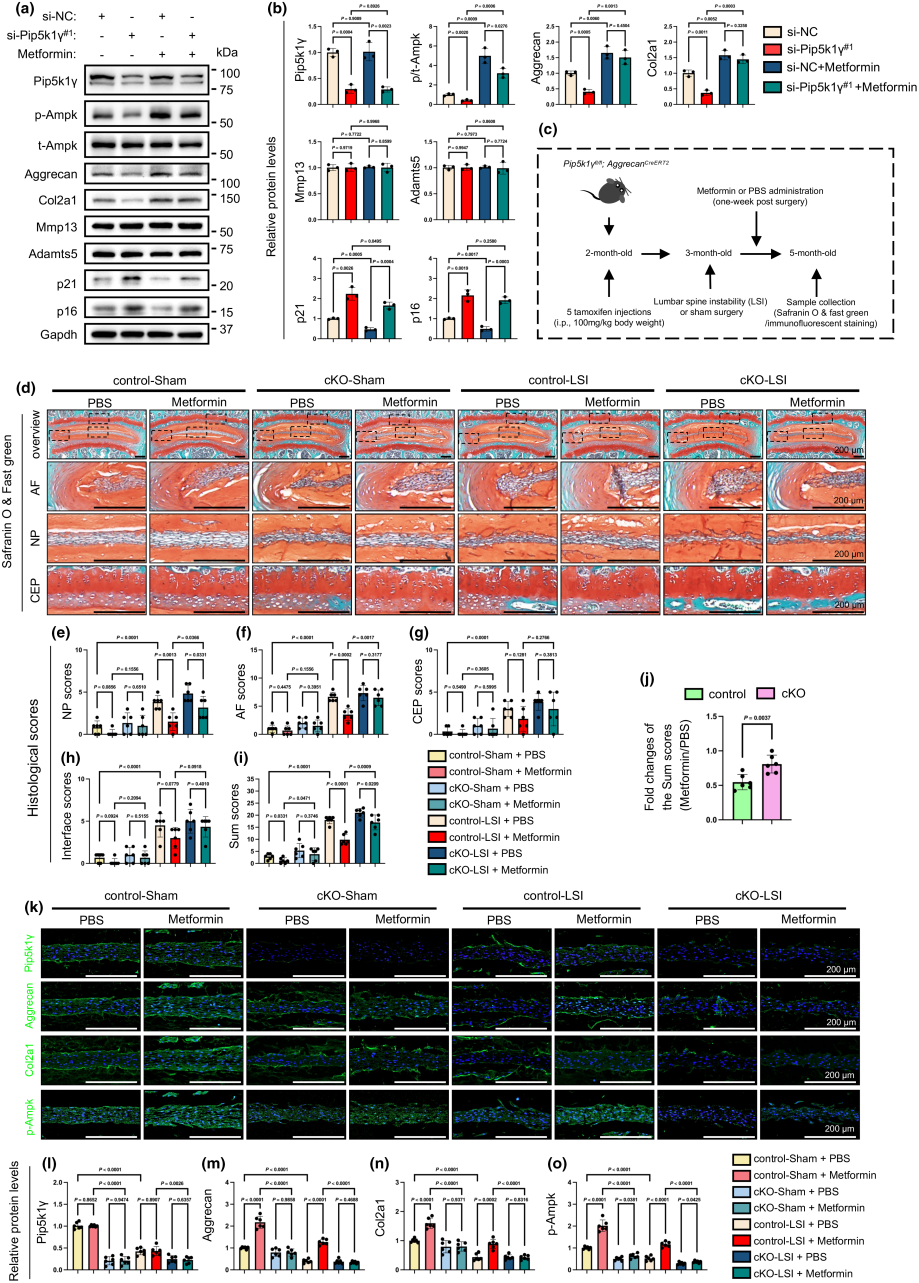
Effects of Pip5k1γ deletion on therapeutic effects of metformin in LSI‐induced disc degeneration in mice. (a, b) NP cells were transfected with si‐NC or si‐Pip5k1γ^#1^ for 48 h and then treated with metformin for another 24 h. Total protein extracts were analyzed by western blotting using antibodies against Pip5k1γ, t‐Ampk, p‐Ampk, aggrecan, Col2a1, Mmp13, Adamts5, p21, and p16. Gapdh was used as a loading control. Quantitative data are shown in (b). *N* = 3 for each group. (c) A time‐course scheme illustrating the experimental design and time points of the metformin treatment experiment. At 2 month of age, male *Pip5k1γ* ^*fl*/*fl*^; *Aggrecan* ^*CreERT2*^ mice were injected with TAM to induce *Pip5k1γ* deletion. One month later, the mice underwent either lumbar spine instability (LSI) or sham surgery to induce disc degeneration. Metformin administration started one week after the surgery, with PBS (the solvent of metformin) used as the control for metformin treatment. All mice were sacrificed at 5 months of age and the L4‐5 lumbar discs were collected for further analysis. (d) Safranin O and fast green staining displaying the structure of lumber discs from different groups. Higher magnification images of AF, NP, and CEP areas (black dashed boxes) are shown in the lower panels. Scale bar: 200 μm. (e–i) Evaluation of NP scores (e), AF scores (f), CEP scores (g), Interface scores (h), and Sum scores (i). *N* = 6 for each group. (j) Fold changes of the Sum scores in metformin‐treated control and cKO mice after LSI surgery. The data was normalized to corresponding PBS‐treated controls. *N* = 6 for each group. (k–o) Quantitative IF analyses of the protein expressions of Pip5k1γ, aggrecan, Col2a1, and p‐Ampk in NP tissues. *N* = 6 for each group. Results are expressed as mean ± standard deviation (SD). *p*‐values for individual analyses are provided.

We next investigated whether the NP cell phenotypes induced by Pip5k1γ loss could be reversed by metformin, a well‐known Ampk activator (Meng et al., 2015). NP cells were transfected with either si‐NC or si‐Pip5k1γ in the presence or absence of 500 μM metformin. This concentration of metformin was set based on prior research by Xian et al. (2021), which demonstrated its effectiveness in up‐regulating Ampk phosphorylation without compromising cell viability (Figure S2). The results showed that metformin treatment significantly upregulated the expression levels of p‐Ampk and anabolic ECM proteins in both si‐NC‐ and si‐Pip5k1γ‐treated NP cells, while the levels of ECM catabolic proteins were not markedly altered (Figure 6a,b). Furthermore, the increases in expression of p21 and p16 proteins induced by Pip5k1γ loss were slightly reduced after metformin treatment (Figure 6a,b).

Furthermore, we explored the therapeutic potential of metformin in the lumbar spine instability (LSI)‐induced disc degeneration in both Pip5k1γ‐intact (control) and Pip5k1γ‐deficient (cKO) mice. Two‐month‐old *Pip5k1γ* ^*fl*/*fl*^; *Aggrecan* ^*CreERT2*^ male mice underwent TAM/corn oil treatment, followed by LSI or sham operations 1 month later as we previously reported (Chen, Wu, et al., 2022; Wu et al., 2023). Metformin was administrated (dissolved in drinking water, 205 mg/kg body weight) starting 1 week post LSI surgery for an additional 2 months, with PBS used as a control treatment for metformin, as detailed in Figure 6c. The concentration and duration of metformin treatment were referenced from the work of Li et al (2020), where this regimen was reported to effectively up‐regulate the phosphorylation of Ampk in vivo (Li et al., 2020). Histology analysis revealed that LSI significantly elevated the degeneration‐related histological scores, including the NP scores, AF scores, CEP scores, Interface scores, and Sum scores, in both control and cKO mice (Figure 6d–i). While metformin effectively reduced most histological scores in control mice, it only lowered the NP scores and Sum scores in cKO mice compared to PBS‐treated groups (Figure 6d–i). Notably, the fold changes of total histological scores (metformin‐treated/PBS‐treated), were significantly higher in cKO mice than those in control mice (Figure 6j). More importantly, metformin treatment failed to up‐regulate the expression levels of aggrecan, Col2a1, and p‐Ampk, in LSI‐treated cKO group when compared with its effects on these molecules in control mice (Figure 6k–o). Collectively, these findings suggest that Pip5k1γ deficiency results in a limited therapeutic benefit of metformin in mitigating LSI‐induced disc lesions in mice.

## 4 DISCUSSION

The degeneration of intervertebral discs is a naturally occurring process as part of the aging program (Hemanta et al., 2016; Schoenfeld et al., 2011). However, the complex molecular mechanism of aging‐induced disc degeneration remains poorly understood. In this study, we demonstrate that Pip5k1γ expression is important for maintaining disc homeostasis and protection against aging‐related disc degeneration. We find that genetic ablation of Pip5k1γ results in multiple severe degenerative defects, including the loss of NP cells, decreased ECM anabolism, and spontaneous NP fissures in the lumbar discs of aged mice. More importantly, the degenerative defects induced by Pip5k1γ loss highly mirror those of aging‐induced disc degeneration lesions, suggesting that Pip5k1γ loss may play an important role in aging‐related DDD. In accordance with this study, our previous study showed that Pip5k1γ loss induced cartilage degradation and osteoarthritic lesions in the knee joints in aged mice but not in adult mice (Qu et al., 2022). Interestingly, in both cartilage and disc tissues, we demonstrate that Pip5k1γ deletion causes multiple aging‐like spontaneous tissue damages. This study reveals a vital role of Pip5k1γ in control of disc homeostasis in the context of aging.

The ECM stands as a critical component for the functional and structural maintenance of intervertebral discs. Throughout life, the ECM undergoes constant turnover in both content and quality, ensuring disc integrity and flexibility (Bonnans et al., 2014; Zhang et al., 2022). A homeostatic ECM network is not only essential for the formation and biomechanical reinforcement of disc tissues but also offers a unique microenvironment for maintaining a healthy phenotype of disc cells (Aszódi et al., 1998; Attia et al., 2011; Boyd et al., 2008; Bridgen et al., 2013). Recent studies have underscored the importance of ECM in the regulatory mechanisms of disc homeostasis and disease. On one hand, pathogenic factors, such as aging, inflammation, and aberrant mechanical stress, reduce the production of anabolic ECM proteins and induces excessive expression of catabolic ECM enzymes (Chen, Wu, et al., 2022; Lai et al., 2022; Li et al., 2022; Wang et al., 2020). On the other hand, altered ECM composition further amplifies the pathological changes in disc cells, thus forming a vicious cycle (Buser et al., 2014; Chen et al., 2017; Feng et al., 2015, 2017). Yet, how ECM composition is precisely regulated by disc cells during homeostatic and disease conditions remains to be investigated. In this study, we find that genetic deletion of Pip5k1γ expression in disc cells abolishes the synthesis of aggrecan and Col2a1, both components of anabolic ECM, without affecting the production of catabolic enzymes. Notably, our recent study demonstrated that Pip5k1γ deletion in chondrocytes also depressed ECM anabolism without upregulating ECM catabolism (Qu et al., 2022). These findings reveal a possible common mechanism by which Pip5k1γ regulates ECM homeostasis in both chondrocytes and NP cells.

The senescence of NP cells is a major pathological mechanism of disc degeneration (Ashraf et al., 2021; Patil et al., 2018). Clinical and animal studies have shown the accumulation of senescent NP cells in aged intervertebral discs, characterized by increased SA‐β‐Gal‐staining (Veroutis et al., 2021). Activation of cellular senescent pathways, such as the p53/p21/RB and p16‐RB pathways, promoted NP cell apoptosis and disc degeneration (Feng et al., 2016, 2018; Sun et al., 2021; Wang, Cai, et al., 2016; Zhang et al., 2021). Moreover, senescent NP cells can create a detrimental extracellular milieu, called the senescence‐associated secretory phenotype, leading to advanced inflammation and ECM degradation (Wu, Shen, et al., 2022). Recently, several studies have suggested that NP cell senescence is a promising therapeutic target for treating DDD. For instance, senolytic drugs, such as dasatinib and quercetin, eliminated senescent NP cells and attenuated degenerative disc defects in rodent models (Novais et al., 2021; Shao et al., 2021). Dehydrocostus lactone and morroniside demonstrated beneficial effects on ameliorating disc degeneration by suppressing NP cell senescence (Chen et al., 2021; Zhou et al., 2022). Despite the crucial role of NP cell senescence in disc degeneration, the physiological signals that inhibit NP cell senescence remain elusive. In this study, we demonstrate that Pip5k1γ loss significantly increased the SA‐β‐Gal‐staining staining and the expression levels of p53, p21, p16, Rb proteins, all well‐known cellular senescence markers, in NP cells. These important findings suggest that the expression of Pip5k1γ is critical for maintaining a non‐senescent status of NP cells. Whether overexpression and activation of Pip5k1γ can prevent or reverse NP cell senescence in the context of disc degeneration needs to be determined in future studies.

The Ampk protein is a trimeric enzyme comprising a catalytic α subunit and two regulatory β‐ and γ‐subunits (Carling, 2017). In mammalian cells, the Ampk acts as a key intracellular energy sensor and is activated upon energy disequilibrium (Hardie, 2007, 2014; Marsin et al., 2000; Russell 3rd et al., 2004; Salt et al., 1998). It is well‐known that the activation of Ampk is regulated through three major mechanisms, including increased AMP/adenosine diphosphate (ADP) ratio, liver kinase B1 (LKB1) signal activation, and calcium signaling cascade (Mihaylova & Shaw, 2011). Once activated, the Ampk induces the transcription of peroxisome proliferator‐activated receptor‐g co‐activator 1 alpha (PGC1α) to promote mitochondrial biogenesis and functions, which increases energy supply and restores cellular homeostasis (Herzig & Shaw, 2018). Recent studies have highlighted the importance of the Ampk pathway in the pathological mechanism of DDD (Wang et al., 2021). Healthy NP cells express a high level of p‐Ampk, whereas decreased p‐Ampk level was observed in the degenerative disc specimens from patients and animal models (Liu et al., 2019; Song et al., 2018). Cumulative evidence reveals that the activation of Ampk is crucial for preventing cellular senescence and tissue degeneration (Han et al., 2016; Li et al., 2020). Ampk activators, such as metformin and resveratrol, show protective effects against DDD through inhibiting NP cell senescence (Chen et al., 2016; Ren et al., 2022; Wang, Zhu, et al., 2016). Our previous study demonstrated that Pip5k1γ loss suppressed CaMKII phosphorylation and Ca^2+^ influx in the mesenchymal stem cells (Yan et al., 2022). In this study, we find that Pip5k1γ deletion significantly downregulates the phosphorylation of CaMKII, Ca^2+^ influx, and the activation of Ampk, leading to increased cellular senescence of NP cells. Activating the Ampk inhibits NP cell senescence induced by Pip5k1γ loss. These results suggest a novel pathway, which contains Pip5k1γ, CaMKII, and Ampk, modulates NP cell homeostasis and senescence. Whether abnormalities in the Pip5k1γ‐CaMKII‐Ampk pathway play an important role in the pathogenesis of human DDD warrants further investigation.

Results from this study highlight an intracellular signaling network involving Pip5k1γ, CaMKII, Ampk, and ECM metabolism. By examining both the mRNA and protein levels of these key molecules, we found that the absence of Pip5k1γ significantly reduces both the mRNA and protein levels of anabolic factors aggrecan and Col2a1, while not affecting the mRNA and protein levels of catabolic or anti‐catabolic factors, such as Mmp13, Adamts5, and Timp. Interestingly, although at the protein level, Pip5k1γ deficiency markedly increased the expression of senescence‐associated markers and depressed the activation of the CaMKII‐Ampk pathway, we did not observe any significant changes in the mRNA levels of these proteins. These findings suggest that Pip5k1γ likely facilitates the production of anabolic extracellular proteins at the transcriptional level. In contrast, its absence seems to inhibit CaMKII‐Ampk activation and contribute to NP cell senescence via post‐transcriptional mechanisms. The molecular mechanisms underlying Pip5k1γ regulation of this network warrant further investigation.

Metformin, primarily recognized as an anti‐diabetic drug, has emerged as a potential treatment for degenerative skeletal diseases, such as osteoporosis and osteoarthritis (Bahrambeigi et al., 2019; Chen, Gan, et al., 2022; Feng et al., 2020; Li et al., 2020; Song et al., 2022). Recent studies suggest metformin as a promising therapeutic agent in treating DDD. For instance, Liao et al. reported that extracellular nanovesicles derived from metformin‐treated MSCs ameliorated disc cell senescence both in vitro and in vivo (Liao et al., 2021). Furthermore, Ramanathan and coworkers highlighted the anti‐inflammatory properties of metformin on intervertebral disc cells (Ramanathan et al., 2022). Another study demonstrated that metformin could activate Ampk in NP cells in a dose‐ and time‐dependent manner. This effect helps ameliorate disc degeneration by protecting nucleus pulposus cells against apoptosis and senescence (Chen et al., 2016). Metformin also protected against NP cell senescence through inactivating the cGAS‐STING pathway (Ren et al., 2022). Han et al. reported that metformin has the potential to reduce lipopolysaccharide‐induced inflammation in disc cells, possibly by inhibiting HMGB1 release (Han et al., 2019). The activation of the Ampk pathway is a common thread in the protective effects of metformin against DDD. Results from this study reveal that Pip5k1γ expression is crucial for maintaining the Ampk activation in NP cells and loss of Pip5k1γ largely compromised the therapeutic efficacy of metformin on mitigating disc lesions in mouse model. Thus, maintaining a physiological level of Pip5k1γ expression in NP cells seems to be critical for achieving a high therapeutic efficacy of metformin treatment.

We acknowledge several limitations of this study. First, it should be noted that the *Aggrecan* ^*CreERT2*^ transgene is effective in all disc tissues, including NP, AF, and CEP (Zheng et al., 2019). Although Pip5k1γ is predominately expressed in NP cells, it is also expressed in cells of AF and CEP. In comparing with the control group, we also observed some mild pathological changes, such as decreased cellularity, loss of anabolic ECM proteins, and increased apoptotic activity in AF and CEP tissues of cKO discs. It remains unclear whether the defects in these areas were due to local loss of Pip5k1γ or indirect effects from Pip5k1γ deletion in NP cells. Second, the human NP tissues were obtained from patients who underwent unilateral biportal endoscopic discectomy, an emerging minimally invasive spinal surgery. This operation offers advantages, such as minimal trauma, reduced bleeding, rapid recovery, fewer complications, and precise efficacy. However, accessing other disc tissues, such as the AF and CEP, through this surgery is challenging. Consequently, in this study, we were only able to examine the expression patterns of Pip5k1s in human NP tissues. Thirdly, whether the loss of Pip5k1γ expression could contribute to the pathology of human disc degeneration requests further validations. Fourthly, we did not test whether overexpressing Pip5k1γ in disc tissues can reverse or delay aging‐ or LSI‐induced disc degeneration.

## AUTHOR CONTRIBUTIONS

Study design: GX, XW and MC. Study conduct and data collection: MC, FL, MQ, XJ, TH, SH, QY and LW. Data analysis: GX, XW and MC. Data interpretation: GX, XW, MC and DC. Drafting the manuscript: GX and XW. GX, XW and MC take the responsibility for the integrity of the data analysis.

## CONFLICT OF INTEREST STATEMENT

The authors declare that they have no competing financial interests.

## Supporting information


Figures S1‐S3.

Tables S1‐S3.

## Data Availability

All data generated for this study are available from the corresponding authors upon reasonable request.
