# Supplementary material for: Pip5k1γ promotes anabolism of nucleus pulposus cells and intervertebral disc homeostasis by activating CaMKII‐Ampk pathway in aged mice
Source: Aging Cell. 2024 Jun 5;23(9):e14237. doi: 10.1111/acel.14237 (PMC11488325; doi:10.1111/acel.14237)
Supplement: Supplementary file 1 — Figures S1‐S3. [file ACEL-23-e14237-s002.pdf]

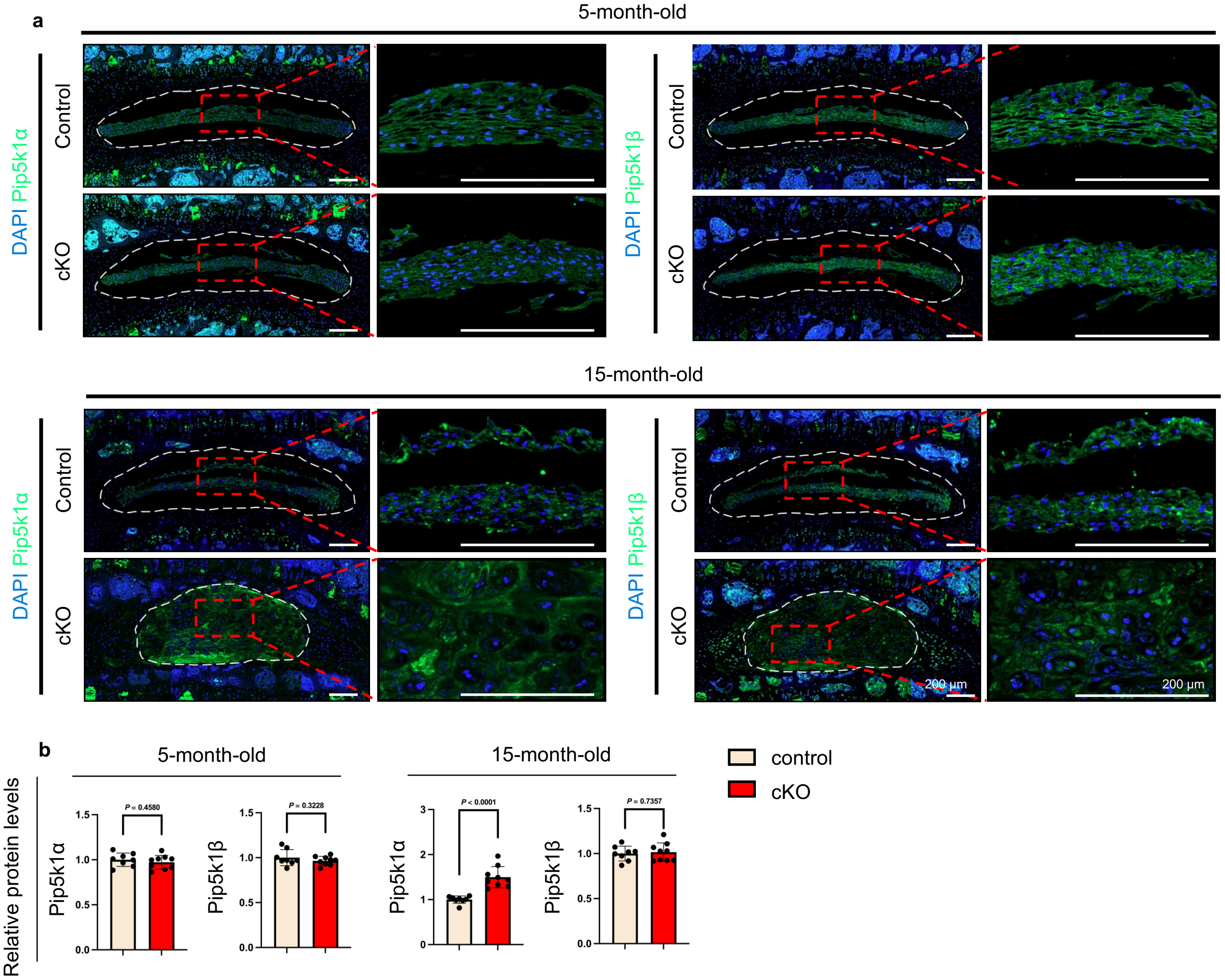

Supplementary Figure 1: Expression of Pip5k1α and Pip5k1β in NP tissues of Pip5k1γ cKO mice. (**a**) Representative IF staining showing the expressions of Pip5k1α and Pip5k1β in lumbar disc sections from control and cKO mice at 5 or 15 months of age. Higher magnification images of the NP tissues (red dashed boxes) are shown in the right panels. Scale bar: 200 μm. (**b**) Percentages of positively stained cells in the NP tissues. *N* = *8* for control and *N* = *9* for cKO. Results are expressed as mean ± standard deviation (s.d.). P-values for individual analyses are provided. NP: nucleus pulposus.

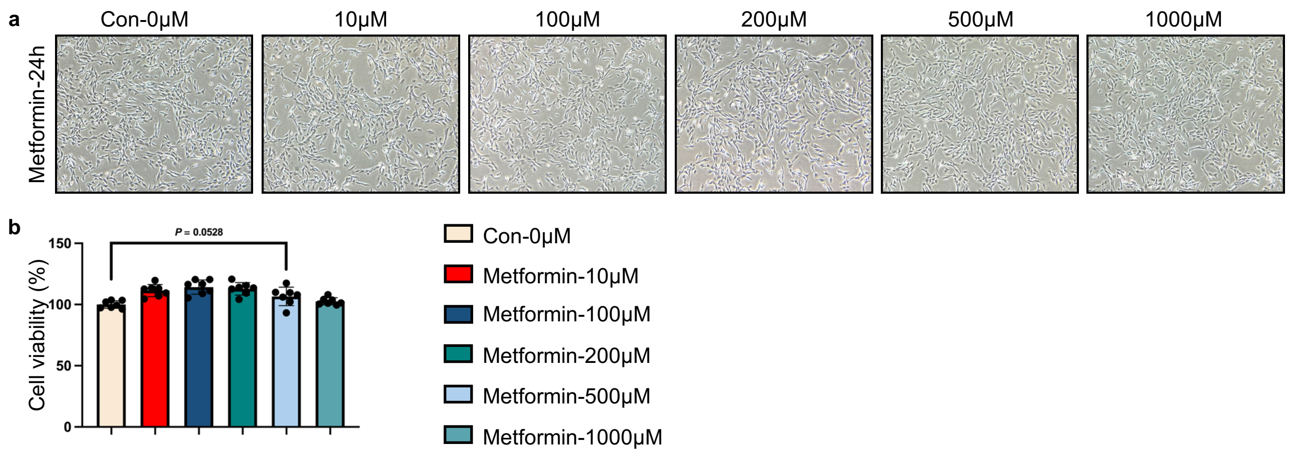

Supplementary Figure 2: Metformin treatment does not affect the viability of NP cells in vitro. (**a**) Representative microscopic images of NP cells treated with different concentrations of metformin for 24 hours in vitro. (**b**) Cell viability of NP cells determined by the cell counting kit 8 (CCK-8) assay. *N* = *7* for each group. Results are expressed as mean ± standard deviation (s.d.). P-values for individual analyses are provided. NP: nucleus pulposus.

Cell types	NP cell line			ATDC5 chondrogenic cell line			MLO-Y4 osteocyte cell line		
Genes	Cq values								
Vimentin	17.86	17.71	17.64	18.35	18.27	18.15	17.16	17.10	17.10
Laminin	27.38	27.32	27.30	36.26	36.19	36.00	37.09	34.93	40.79
Basp1	28.23	28.15	28.16	30.51	30.59	30.51	31.66	31.70	32.25
Tbxt	28.78	28.85	28.58	34.71	35.50	35.30	31.59	31.64	31.18
CD24	23.87	23.93	23.92	25.96	25.91	25.64	38.10	29.94	33.32
Gapdh	19.75	19.86	19.73	17.11	17.10	17.10	18.46	18.46	18.15

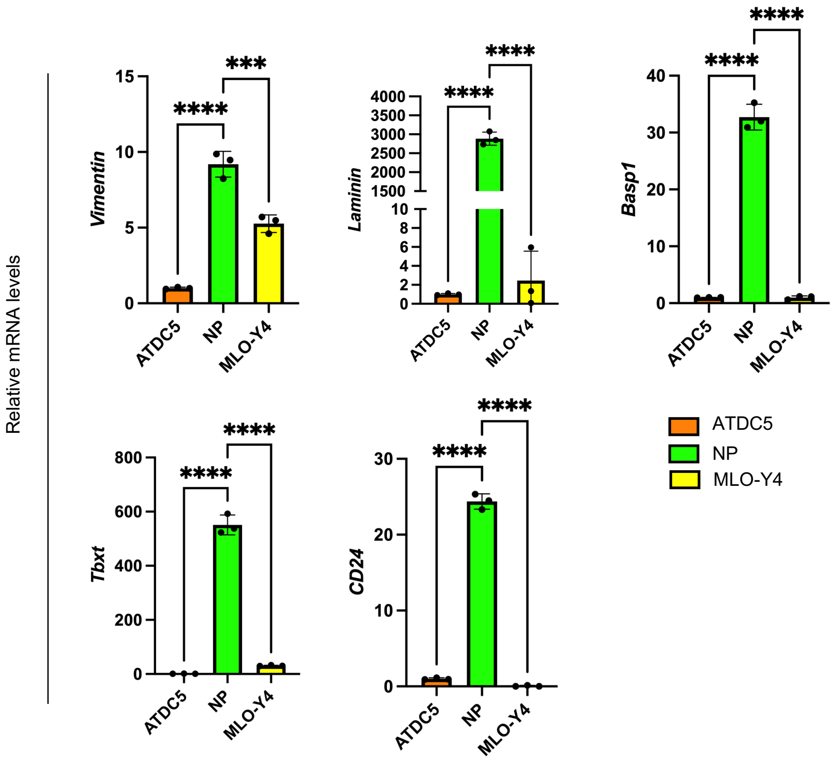

Supplementary Figure 3: Validation of the phenotypes of the NP cell line. (**a**) Cq values of marker genes of NP cells, including *vimentin*, *laminin*, *Basp1*, *Tbxt*, *CD24*, determined by real time PCR analysis. A chondrogenic ATDC5 cell line and a MLO-Y4 osteocyte cell line were used as a non-NP control. Gapdh gene served as a housekeeping gene. (**b**) Quantitative analysis of relative mRNA levels of above markers. *N = 3* for each group. Results are expressed as mean ± standard deviation (s.d.). P-values for individual analyses are provided. NP: nucleus pulposus.
