# Supplementary material for: Pip5k1γ promotes anabolism of nucleus pulposus cells and intervertebral disc homeostasis by activating CaMKII‐Ampk pathway in aged mice
Source: Aging Cell. 2024 Jun 5;23(9):e14237. doi: 10.1111/acel.14237 (PMC11488325; doi:10.1111/acel.14237)
Supplement: Supplementary file 2 — Tables S1‐S3. [file ACEL-23-e14237-s001.pdf]

Supplementary Tables

Supplementary Table 1: Patient information.

Case no.	Age (years)	Gender	Disc level	Pfirrmann grading
**Case 1**	29	M	L4/5	II
**Case 2**	46	M	L5/S1	II
**Case 3**	35	F	L4/5	II
**Case 4**	43	F	L5/S1	III
**Case 5**	55	M	L4/5	III
**Case 6**	73	F	L4/5	III
**Case 7**	61	M	L4/5	III
**Case 8**	37	M	L5/S1	III
**Case 9**	72	M	L4/5	IV
**Case 10**	48	F	L4/5	IV
**Case 11**	53	M	L5/S1	IV
**Case 12**	49	F	L5/S1	IV
**Case 13**	62	M	L4/5	V
**Case 14**	59	F	L5/S1	V
**Case 15**	83	M	L5/S1	V
**Case 16**	71	M	L4/5	V

Supplementary Table 2: Antibody information.

Antibody	Company	Catalog #	Application/Dilution
**Pip5k1γ**	Santa Cruz	sc-377061	WB (1:1000); IF (1:100)
**Pip5k1α**	Proteintech	15713-1-AP	WB (1:1000); IF (1:100)
**Pip5k1β**	ABclonal	A7749	WB (1:1000); IF (1:100)
**aggrecan**	ABclonal	A8536	WB (1:1000)
**aggrecan**	Abcam	ab36861	IF (1:100)
**Col2a1**	ABclonal	A1560	WB (1:1000)
**Col2a1**	Sant Cruz	sc-52658	IF (1:100)

**Mmp13**	Abcam	ab39012	WB (1:1000); IF (1:100)
**Adamts5**	Abcam	ab41037	WB (1:1000); IF (1:100)
**Ki67**	CST	12202	IF (1:100)
**active caspase 8**	CST	9429	IF (1:100)
**active caspase 3**	Sigma-Aldrich	C8487	IF (1:100)
**p21**	CST	2947	WB (1:1000); IF (1:100)
**p16**	Abcam	ab211542	WB (1:1000); IF (1:100)
**p53**	CST	2524	WB (1:1000)
**RB**	Abcam	ab181616	WB (1:1000)
**t-Erk**	CST	9102	WB (1:1000)
**p-Erk**	CST	9101	WB (1:1000)
**t-Creb**	CST	9197	WB (1:1000)
**p-Creb**	CST	9198	WB (1:1000)
**t-Ampk**	CST	2532	WB (1:1000); IF (1:100)
**p-Ampk**	CST	2531	WB (1:1000); IF (1:100)
**t-CaMKII**	CST	4436	WB (1:1000); IF (1:100)
**p-CaMKII (T286)**	CST	12716	WB (1:1000); IF (1:100)
**Timp-1**	Proteintech	16644-1-AP	WB (1:1000); IF (1:100)
**Timp-3**	Abcam	ab39184	WB (1:1000); IF (1:100)

Supplementary Table 3: Effect of Pip5k1γ siRNA knockdown on Gapdh mRNA levels in NP cells in vitro. NP cells were transfected with negative control siRNA (si-NC) or Pip5k1γ-targeting siRNAs for 48 hours. The total RNA was extracted and subjected to real-time polymerase chain reaction assays to determine the mRNA levels of Gapdh. The results indicated that the mRNA levels of Gapdh, as revealed by the Cq values, remained unchanged following the knockdown of Pip5k1γ.

	**si-NC**			**si-Pip5k1γ #1**			**si-Pip5k1γ #2**			**si-Pip5k1γ #3**		
**Repeat 1**	17.64	17.68	17.59	17.51	17.44	17.38	17.24	17.27	17.32	17.49	17.54	17.58
**Repeat 2**	19.56	19.79	19.77	19.53	19.37	19.26	19.56	19.41	19.35	19.78	19.56	19.50
**Repeat 3**	19.39	19.33	19.34	19.48	19.44	19.31	19.51	19.53	19.39	19.33	19.30	19.16
	**Cq values of *Gapdh* mRNA**											
